# Vaginal Tumor Cell Exfoliation in Cervical and Endometrial Cancer: A Comparative Washing Cytology Study with Implications for Minimally Invasive Surgery

**DOI:** 10.3390/jcm14207383

**Published:** 2025-10-19

**Authors:** Jung Min Ryu, Youn Seok Choi, Sun-Jae Lee, Yoon Young Jeong

**Affiliations:** 1 Department of Obstetrics and Gynecology, School of Medicine, Daegu Catholic University, Daegu 42472, Republic of Korea; medgirl1231@cu.ac.kr (J.M.R.); drcys@cu.ac.kr (Y.S.C.); 2 Department of Pathology, School of Medicine, Daegu Catholic University, Daegu 42472, Republic of Korea; pathosjlee@cu.ac.kr

**Keywords:** vaginal washing cytology, tumor cell exfoliation, cervical and endometrial cancer, minimally invasive surgery

## Abstract

**Background/Objectives**: Minimally invasive surgery (MIS) is widely used for gynecologic malignancies, but the LACC trial reported significantly worse survival in early-stage cervical cancer compared with open surgery, raising concerns about its oncologic safety. Tumor cell spillage during intracorporeal colpotomy in the Trendelenburg position has been proposed as a potential mechanism underlying these findings. This study aimed to assess the presence of tumor cells exfoliated into the vaginal cavity in cervical and endometrial cancers using vaginal washing cytology. **Methods**: We retrospectively analyzed patients newly diagnosed with cervical or endometrial cancer between June 2021 and February 2025. Vaginal washing cytology was performed before treatment and interpreted. Chi-square or Fisher’s exact tests and multivariate logistic regression were used to identify factors associated with positive cytology. **Results**: Positive cytology detected more often in cervical than in endometrial cancer (all stages: 33.3% [12/36] vs. 6.5% [3/46], *p* = 0.002; early stage: 24.0% [6/25] vs. 0% [0/38], *p* = 0.003). Multivariate analysis confirmed cervical cancer (OR 14.24, 95% CI 1.83–110.89, *p* = 0.011) and FIGO stage III–IV (OR 9.53, 95% CI 2.08–43.61, *p* = 0.004) as independent predictors. **Conclusions**: Tumor cells exfoliated into the vagina were significantly more frequent in cervical cancer, supporting a mechanism by which intracorporeal colpotomy may allow peritoneal entry. Further studies should reassess MIS for early-stage cervical cancer, considering transvaginal colpotomy in the horizontal position.

## 1. Introduction

Cervical cancer is a malignant tumor arising in the cervix, the anatomical region connecting the uterus and the vagina. It is strongly associated with infection by high-risk human papillomavirus (HPV) genotypes, particularly HPV-16 and HPV-18, which play a key role in carcinogenesis [1,2]. Endometrial cancer, by contrast, originates from the endometrial lining of the uterus, with known risk factors including estrogen imbalance, obesity, hypertension, and diabetes mellitus [3,4].

Both cervical and endometrial cancers are typically treated surgically in early stages. Surgical approaches include minimally invasive surgery (MIS; laparoscopic or robotic) as well as open laparotomy. MIS offers several advantages over open surgery, including reduced scarring, less postoperative pain, and faster recovery.

In endometrial cancer, large randomized controlled trials such as the GOG LAP2 and LACE trials demonstrated equivalent oncologic outcomes between laparoscopic and open approaches [5,6].

In cervical cancer, retrospective studies conducted before the LACC trial (2018) reported no significant prognostic difference between laparoscopic and open approaches [7,8]. However, the LACC trial revealed that MIS was associated with significantly worse oncologic outcomes compared with open surgery [9]. Consequently, open surgery has become the preferred approach for early-stage cervical cancer in many institutions. Since survival outcome is the highest priority in cancer treatment, the decision to abandon MIS and continue with open surgery has become a clinically significant issue, raising important questions about balancing oncologic safety with patient-centered benefits.

Several mechanisms have been proposed to explain the inferior oncological outcomes of MIS in cervical cancer. The use of uterine manipulators and carbon dioxide (CO_2_) insufflation during laparoscopic procedures may contribute to tumor spillage or dissemination of malignant cells [9,10]. However, these mechanisms are less convincing in endometrial cancer, where no significant difference in oncologic outcomes has been observed between MIS and open surgery. Furthermore, compared to transvaginal colpotomy, intracorporeal colpotomy has been reported to increase the risk of positive vaginal cuff margins and intraperitoneal tumor dissemination in early-stage cervical cancer patients undergoing MIS radical hysterectomy [11].

**Figure 1:**
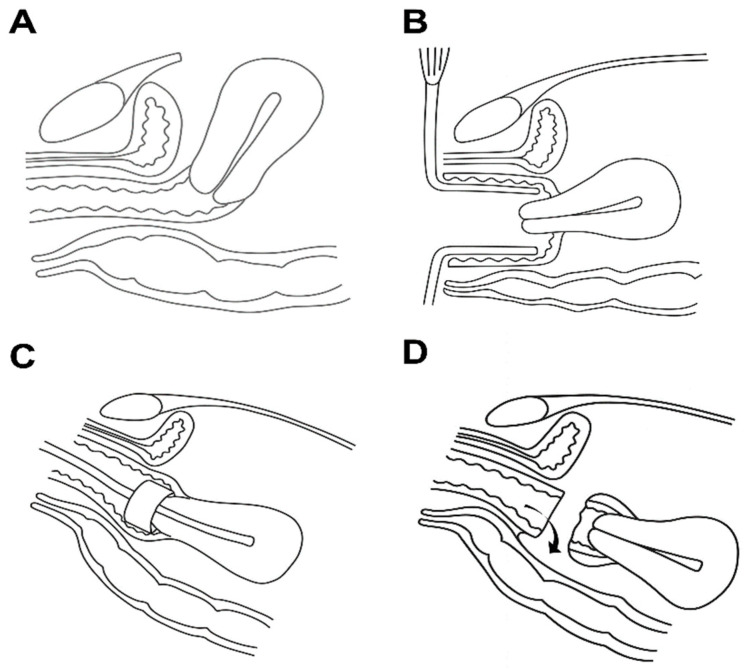
Schematic illustration of colpotomy during hysterectomy under different surgical approaches and patient positions. (**A**) Transabdominal hysterectomy: The patient is placed in the supine position. During colpotomy, the uterus is pulled ventrally, resulting in the vagina being aligned close to a horizontal axis. (**B**) Minimally invasive surgery with transvaginal colpotomy: While the patient remains in the lithotomy position, the Trendelenburg tilt used during most of the procedure is adjusted to a horizontal table position for colpotomy, placing the vagina close to a horizontal axis. (**C**) Minimally invasive surgery with intracorporeal colpotomy: With the patient in the lithotomy and Trendelenburg position, the upper vagina becomes inclined into the abdominal cavity, and a uterine manipulator is usually employed during this procedure. (**D**) During minimally invasive surgery with intracorporeal colpotomy under lithotomy and Trendelenburg position: Because the upper vagina tilts into the pelvic cavity, spillage of vaginal contents into the peritoneal cavity may occur.

Considering these factors, the surgical position (Trendelenburg position) and the method of colpotomy may play a role in oncologic safety. In open surgery (transabdominal surgery), the uterus is typically pulled anteriorly, and colpotomy is performed along a nearly horizontal vaginal axis, which may reduce the likelihood of vaginal content entering the peritoneal cavity (Figure 1A). A similar principle applies to transvaginal colpotomy during MIS (Figure 1B). In contrast, intracorporeal colpotomy is performed in the Trendelenburg position, where the upper vagina is tilted toward the peritoneal cavity, potentially increasing the chance of intraperitoneal spillage of vaginal contents (Figure 1C,D).

Based on these considerations, we hypothesized that the surgical approach and colpotomy method could influence the risk of tumor cell dissemination. To explore this possibility, we performed vaginal washing cytology for cervical and endometrial cancers in order to directly investigate the presence of exfoliated tumor cells within the vaginal cavity.

## 2. Materials and Methods

Patients newly diagnosed with cervical or endometrial cancer at Daegu Catholic University Hospital between June 2021 and February 2025 were enrolled. Patients with recurrent disease, prior pelvic or abdominal surgery for other malignancies, or previous pelvic/abdominal radiation therapy were excluded. Cancer staging was performed according to the International Federation of Gynecology and Obstetrics (FIGO) 2018 classification for cervical cancer and the FIGO 2009 classification for endometrial cancer.

Cervical cancer was diagnosed by cervical punch biopsy or conization, and endometrial cancer by endometrial biopsy. All patients underwent gynecologic examination, pelvic magnetic resonance imaging (MRI), and positron emission tomography–computed tomography (PET-CT) as part of the initial evaluation. Pelvic MRI findings suggestive of lymph node metastasis included nodes > 10 mm in short-axis diameter, irregular margins, and heterogeneous signal intensity. On PET-CT, metastatic lymph nodes or distant lesions were defined as areas showing increased ^18F-fluorodeoxyglucose (FDG) uptake, with a maximum standardized uptake value (SUVmax) typically ≥2.5 and higher than that of adjacent normal tissue.

Each patient’s clinical characteristics, including age, body mass index (BMI), parity, stage, metastasis, final histology, tumor grade, treatment, tumor marker and surgery type (MIS or Open) were retrospectively collected from medical records. Tumor marker obtained within before surgery were analyzed.

Vaginal washing cytology was performed during the diagnostic work-up. With the patient in the lithotomy position, the vagina was dilated using a speculum and any vaginal discharge or blood was not removed prior to the procedure. Approximately 20 mL of 0.9% normal saline was instilled into the vaginal cavity. The saline was allowed to remain in the vaginal cavity for approximately 10 s before being collected into a sterile container. The irrigation fluid was then transferred to a clean syringe and subsequently placed into a cytology bottle for further processing and analysis.

Specimens were first gently mixed and aliquoted into cytology centrifuge tubes. They were centrifuged at 2500 rpm for 10 min, and the supernatant was discarded. A single drop of egg albumin was added to the cell pellet, which was then thoroughly mixed. The suspension was transferred (1–2 drops) into a Cytospin chamber assembled in the order of glass slide, filter card, and funnel. The chamber was mounted in a Cytospin centrifuge and spun at 1500 rpm for 2 min. After centrifugation, the funnel and filter card were removed sequentially, and the slide was immediately immersed in a fixative for preservation of the cytologic smear. For preparation of cell blocks, the remaining specimen was treated with 95% alcohol and centrifuged again (2500 rpm for 10 min). The pellet was refrigerated for at least 4 h to allow partial coagulation, then transferred onto a lens paper placed on a cell block cassette using an applicator. The pellet was folded with forceps to fit the cassette size, enclosed securely with the cassette lid, and immersed in 10% neutral buffered formalin. After fixation, all cassettes were sent to the grossing room for routine tissue processing. Thin sections (3–4 μm) were subsequently cut from the formalin-fixed paraffin-embedded (FFPE) blocks and stained with hematoxylin and eosin (H&E) for microscopic evaluation [12].

All cell block specimens were reviewed by a specialized pathologist at Daegu Catholic University Medical Center. Interobserver agreement for the cytologic diagnosis was assessed using Cohen’s kappa statistic, which demonstrated substantial agreement (κ = 0.727, standard error = 0.104; 95% confidence interval, 0.523–0.931). Cytologic interpretation followed The International System for Reporting Serous Fluid Cytology (TIS, 2020), which classifies results as follows: Nondiagnostic, Negative for malignancy, Atypia of undetermined significance (AUS), Suspicious for malignancy (SFM), or Malignant.

Cases were classified into the following categories:

1. *Nondiagnostic*—insufficient cellularity for interpretation;
2. *Negative for malignancy*—no malignant cells identified, only reactive changes;
3. *Atypia of undetermined significance (AUS)*—equivocal cytologic atypia;
4. *Suspicious for malignancy (SFM)*—strongly suggestive of malignancy but not definitive;
5. *Malignant*—unequivocal malignant cells present.

**Figure 2:**
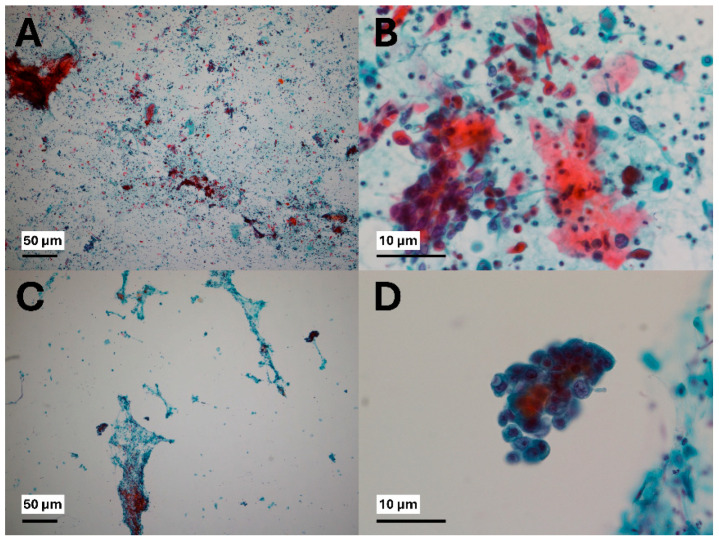
Representative microscopic images of positive vaginal washing cytology cell block specimens stained with hematoxylin and eosin (H&E). (**A**) Malignant cells consistent with squamous cell carcinoma (×40). (**B**) Malignant cells consistent with squamous cell carcinoma (×400). (**C**) Malignant cells consistent with adenocarcinoma (×40). (**D**) Malignant cells consistent with adenocarcinoma (×400).

In this study, *Nondiagnostic* and *Negative for malignancy* categories were regarded as negative cytology results, whereas positive cytology results included *AUS*, *SFM*, and *Malignant* (Figure 2). Following the approach of several previous studies, we classified AUS as a positive cytology result, because the purpose of this study was to determine the presence of abnormal cells in the vaginal cavity [13,14,15].

Data were analyzed using IBM SPSS Statistics (version 25.0; IBM, Armonk, NY, USA). Continuous variables were expressed as mean ± standard deviation (SD), while categorical variables were presented as frequency. Comparisons of categorical variables were conducted using the Chi-square test. Two-tailed *p*-values < 0.05 were considered statistically significant. Odds ratios (ORs) and corresponding 95% confidence intervals (CIs) were calculated using multivariate logistic regression. A post hoc power calculation was performed based on the observed difference in positive cytology rates between groups. This study was conducted in accordance with the Declaration of Helsinki and approved by the Institutional Review Board of Daegu Catholic University Hospital (approval number: DCUMC 2025-06-036). The requirement for written informed consent was waived owing to the retrospective design and the use of anonymized clinical data.

## 3. Results

A total of 82 patients were enrolled, including 36 with cervical cancer and 46 with endometrial cancer. Patient characteristics are summarized in Table 1. The mean age was comparable between the cervical and endometrial cancer groups (55.4 ± 13.9 vs. 55.7 ± 11.8 years, respectively). Among the 36 cervical cancer patients, FIGO 2018 staging classified 15 as stage I, 10 as stage II, 9 as stage III, and 2 as stage IV. Histology included squamous cell carcinoma (*n* = 26), adenocarcinoma (*n* = 8), and others (undifferentiated carcinoma and small cell carcinoma, *n* = 2). 

Among 46 endometrial cancer patients, FIGO 2009 staging were predominantly stage I (35/46), with fewer patients in stages II–IV. Histology included endometrioid carcinoma (*n* = 38), serous carcinoma (*n* = 5), and carcinosarcoma (*n* = 3). Tumor grade also varied; in cervical cancer, most tumors were moderately differentiated (*n* = 31), whereas endometrial cancers included a larger proportion of well-differentiated tumors (*n* = 14). 

Pelvic lymph node metastases were observed in 8 patients in each group, para-aortic lymph node metastases in 4 cervical and 6 endometrial cancer patients, and distant metastases in 1 and 2 patients, respectively. 

Treatment modalities reflected tumor type and stage. In cervical cancer, 16 patients underwent surgery alone, 1 received surgery with adjuvant concurrent chemoradiotherapy (CCRT), 18 received definitive CCRT, and 1 underwent surgery followed by chemotherapy. In endometrial cancer, 26 patients underwent surgery alone, 9 received surgery with radiotherapy, 9 received surgery with chemotherapy, and 2 received surgery followed by both chemotherapy and radiotherapy. Regarding surgical approach, minimally invasive surgery (MIS) was performed in 8 cervical and 25 endometrial cancer patients, while open surgery was performed in 10 cervical and 21 endometrial cancer patients

Vaginal washing cytology was performed in patients with cervical cancer (*n* = 36) and endometrial cancer (*n* = 46) in Table 2. Negative results included cases reported as nondiagnostic or negative for malignancy, although no specimens in our study were classified as nondiagnostic. While positive results encompassed atypia of undetermined significance (AUS), suspicious for malignancy (SFM), or malignant findings. Among all stages, positive cytology was observed significantly more frequently in cervical cancer compared with endometrial cancer (33.3% [12/36] vs. 6.5% [3/46], *p* = 0.002). In the all-stage group, positive cytology in cervical cancer consisted of 7 cases of atypia of undetermined significance (AUS) and 5 cases of Suspicious for malignancy (SFM) or malignant cytology, whereas in endometrial cancer, 2 cases were AUS and 1 case was Suspicious for malignancy (SFM) or malignant. When limited to early-stage disease (stage I or II), positive cytology was detected in 24.0% (6/25) of cervical cancer patients, whereas none of the endometrial cancer patients showed positivity (0/38), demonstrating a significant difference (*p* = 0.003, Fisher’s exact test). In the early-stage group, positive cytology in cervical cancer included 2 cases of AUS and 4 cases of Suspicious for malignancy (SFM) or malignant cytology, while no positive cases were observed in endometrial cancer (0 AUS and 0 Suspicious for malignancy (SFM) or malignant. 

Based on the observed rates (33.3% vs. 6.5%) and sample sizes (*n* = 36, *n* = 46), the post hoc power was approximately 87%.

Multivariate analysis of factors associated with positive cytology is presented in Table 3. Cervical cancer diagnosis (OR 14.24, 95% CI 1.83–110.89, *p* = 0.011) and FIGO stage III–IV (OR 9.53, 95% CI 2.08–43.61, *p* = 0.004) were significant predictors. However, age (<50 vs. ≥50 years) did not reach statistical significance (OR = 0.21, 95% CI = 0.04–1.20, *p* = 0.078). Histologic grade (poor vs. well or moderate) was not significantly associated with cytology positivity (OR = 0.21, 95% CI = 0.06–5.30, *p* = 0.597).

## 4. Discussion

The LACC trial demonstrated significantly worse overall survival and disease-free survival among patients undergoing minimally invasive radical hysterectomy (MIS) for early-stage cervical cancer compared with open surgery [9,16]. In post hoc analyses, recurrence as peritoneal carcinomatosis occurred in 23% of MIS patients versus 9% in open surgery patients, suggesting an increased risk of intraperitoneal dissemination associated with MIS techniques [16].

In contrast, large randomized trials in endometrial cancer, such as GOG LAP2 and LACE, reported no significant difference in prognosis between MIS and open surgery [5,6,17]. This discrepancy raises an important clinical question: what mechanisms underlie the different responses of cervical and endometrial cancers to MIS, and what factors contribute to these differences? The present study was designed to address these questions by directly assessing the exfoliation of tumor cells into the vagina in both cancer types.

Several mechanisms may underlie the inferior outcomes observed in MIS for cervical cancer. First, the use of uterine manipulators has been implicated in adverse oncologic outcomes by facilitating tumor cell dislodgement and peritoneal dissemination [9,16,18]. Multiple studies have reported higher recurrence rates and poorer survival in cervical cancer patients undergoing MIS with manipulator use [19,20]. In endometrial cancer, evidence remains inconsistent; some retrospective analyses found no significant effect on progression-free or overall survival, while other multicenter studies reported higher recurrence rates associated with manipulator use [21,22]. These findings suggest that cervical cancer may be more susceptible to mechanical disruption and tumor spillage than endometrial cancer, likely due to its anatomical proximity to the vaginal canal.

Second, CO_2_ pneumoperitoneum may enhance tumor cell proliferation, motility, and implantation within the peritoneal cavity. Animal and in vitro studies have shown that CO_2_ exposure increases tumor growth and peritoneal metastases compared to gasless conditions [10,23]. However, these effects alone do not fully explain why endometrial cancer outcomes remain unaffected, highlighting that tumor biology and anatomical context play critical roles.

Third, the type of colpotomy performed during MIS appears to be a key factor. Intracorporeal colpotomy prolongs exposure of the cervical stump to CO_2_ pneumoperitoneum, increasing the risk of tumor cell aerosolization and intraperitoneal dissemination [24]. By contrast, transvaginal colpotomy reduces exposure time and may minimize peritoneal seeding [11,25]. Our findings should also be interpreted in light of the surgical approach used for colpotomy. As noted in the Introduction, transvaginal colpotomy during MIS, where the vagina is positioned close to a horizontal axis, which may reduce the risk of spillage. By contrast, intracorporeal colpotomy is performed with the patient in the Trendelenburg position, in which the upper vagina is angled toward the peritoneal cavity, potentially facilitating intraperitoneal spillage of exfoliated cells or other vaginal contents [26,27,28].

In our study, vaginal washing cytology revealed a markedly higher rate of tumor cells exfoliated into the vagina in cervical cancer than in endometrial cancer. When confined to early-stage disease, 24.0% of cervical cancer cases demonstrated tumor cells exfoliated into the vagina, while no cases were observed among patients with endometrial cancer (*p* = 0.003). This pattern suggests that the presence of exfoliated cells in the vaginal cavity is an intrinsic characteristic of cervical cancer, evident even in limited disease.

Multivariate analysis further supported these findings: cervical cancer diagnosis and advanced FIGO stage (III–IV) emerged as independent predictors of detecting tumor cells exfoliated into the vagina, with odds ratios of 14.24 and 9.53, respectively. However, the wide 95% confidence interval for cancer type (1.83–110.89) indicates some uncertainty in the precision of the effect estimate, likely reflecting the modest sample size and the low event rate in endometrial cancer.

Our finding strengthens the hypothesis that anatomic location and biological behavior of cervical tumors facilitate cell release into the vaginal canal. It also provides a potential mechanistic explanation for why surgical techniques that disturb the vaginal axis—particularly during intracorporeal colpotomy in the Trendelenburg position—could increase the risk of intraperitoneal contamination.

Our finding highlights the importance of careful surgical planning. Identifying patients at higher risk of tumor cell dissemination may inform decisions regarding the use of containment strategies, such as protective vaginal closure, and could ultimately improve surgical safety and outcomes [29,30,31]. Indeed, several surgical modifications have been proposed to minimize intraoperative tumor spillage. These include the no-look no-touch technique [32], protective vaginal closure prior to colpotomy [33], and the use of an endobag for specimen retrieval [34], all of which aim to reduce vaginal tumor cell dissemination during MIS for cervical cancer. Incorporating such strategies into surgical practice may help mitigate the risks highlighted by our findings.

The main limitations of this study are its relatively small sample size and single-center, retrospective design, which preclude definitive conclusions regarding prognosis. Moreover, benign gynecologic cases were not included as controls. Given that atypical cells may also appear in inflammatory conditions, the absence of a benign control group is a notable shortcoming. Future large-scale, non-inferiority randomized controlled trials comparing open surgery and minimally invasive surgery with transvaginal colpotomy would be helpful, and such studies are warranted based on the present findings.

## 5. Conclusions

Tumor cells exfoliated into the vagina were significantly more common in cervical cancer than in endometrial cancer. This finding suggests that intracorporeal colpotomy during MIS could allow peritoneal contamination. Future prospective studies are needed to confirm these findings and to evaluate the potential benefits of transvaginal colpotomy performed in a horizontal position during MIS.

## Figures and Tables

**Table 1:** Characteristics of cervical cancer and endometrial cancer patients.

Variables	Cervical Cancer (*n* = 36)		Endometrial Cancer (*n* = 46)	
Age (years) (Mean ± SD)	55.4 ± 13.9		55.7 ± 11.8	
BMI (Mean ± SD)	23.3 ± 3.9		25.1 ± 4.5	
Parity (number)	Nullipara	6	9	
	Primipara	10	4	
	Multipara	20	33	
Stage (number) (Cervical cancer 2018 FIGO stage Endometrial cancer 2009 FIGO stage)	I	15	35	
	II	10	3	
	III	9	6	
	IV	2	2	
Metastasis (number)	Pelvic LN	8	8	
	Para-aortic LN	4	6	
	Distant	1	2	
Histology (number)	SCC	26	Endometrioid	38
	Adenoca	8	Serous	5
	Others	2	Carcinosarcoma	3
Grade (number)	Well	3	Grade 1	14
	Moderate	31	Grade 2	19
	Poor	2	Grade 3	13
Treatment (number)	Surgery	16	Surgery	26
	Surgery + adjuvant CCRT	1	Surgery + RT	9
	CCRT	18	Surgery + CTx	9
	Surgery + CTx	1	Surgery + CTx + RTx	2
Tumor marker (Mean ± SD)	SCC Ag(ng/mL)	9.5 ± 17.1	CA 19-9(U/mL)	21.2 ± 37.7
	CA 125(U/mL)	17.7 ± 12.2.	CA 125(U/mL)	61.2 ± 17.6
Surgery type (number)	MIS	8		25
	Open	10		21

Abbreviations: FIGO, International Federation of Gynecology and Obstetrics; LN, lymph node; SCC, Squamous cell carcinoma; CCRT, concurrent chemoradiotherapy; RT, radiotherapy; CTx, chemotherapy; CA, cancer antigen; MIS, minimally invasive surgery.

**Table 2:** Results of vaginal washing cytology.

	Result	Cervical Cancer	Endometrial Cancer	*p*-Value
All stage Cervical cancer (*n* = 36) Endometrial cancer (*n* = 46)	Negative	24	43	0.002
	Positive	12	3	
Early stage (stage I or II) Cervical cancer (*n* = 25) Endometrial cancer (*n* = 38)	Negative	19	38	0.003 *
	Positive	6	0	

* Fisher’s exact test. Negative = Nondiagnostic or Negative for malignancy. Positive = Atypia of undetermined significance (AUS) or Suspicious for malignancy (SFM) or Malignant.

**Table 3:** Multivariate analysis of factors associated with positive result of vaginal washing cytology.

Value	OR	95% CI	*p*-Value
Age (years) <50 vs. ≥50	0.21	0.04–1.20	0.078
Cancer type Cervical cancer vs. Endometrial cancer	14.24	1.83–110.89	0.011
Stage Stage III or IV vs. stage I or II	9.53	2.08–43.61	0.004
Grade Poor vs. Well or moderate	0.21	0.06–5.30	0.597

Abbreviations: OR, Odds ratio; CI, confidence interval.

## Data Availability

Data are available from the corresponding authors upon reasonable requests.
